# A New Cable-Less Seismograph with Functions of Real-Time Data Transmitting and High-Precision Differential Self-Positioning

**DOI:** 10.3390/s20144015

**Published:** 2020-07-19

**Authors:** Kang Liu, Qingyu You, Juan Wang, Xiqiang Xu, Pengcheng Shi, Kaoshan Dai, Zhenhua Huang, Shiquan Wang, Yuanfeng Shi, Zhibin Ding

**Affiliations:** 1 State Key Laboratory of Marine Geology, Tongji University, Shanghai 200092, China; liukang19@tongji.edu.cn; 2 Key Laboratory of Petroleum Resources Research, Institute of Geology and Geophysics, Chinese Academy of Sciences, Beijing 100029, China; qyyou0880@mail.iggcas.ac.cn (Q.Y.); wangjuan@mail.iggcas.ac.cn (J.W.); xuxiqiang@mail.iggcas.ac.cn (X.X.); pengcheng_shi@uri.edu (P.S.); 3 Shanghai Sheshan National Geophysical Observatory, Shanghai Earthquake Agency, Shanghai 200062, China; 4 Graduate School of Oceanography, University of Rhode Island, Kingston, RI 02882, USA; 5 Department of Civil Engineering and Institute for Disaster Management and Reconstruction, Sichuan University, Chengdu 610065, China; wsq@stu.scu.edu.cn (S.W.); shiyuanfeng@scu.edu.cn (Y.S.); zhibin@scu.edu.cn (Z.D.); 6 Key Laboratory of Deep Underground Science and Engineering, Ministry of Education, Sichuan University, Chengdu 610065, China; 7 College of Engineering, University of North Texas, Denton, TX 76201, USA; zhenhua.huang@unt.edu

**Keywords:** cable-less seismograph, passive seismic exploration, differential positioning, real-time

## Abstract

This study developed a new cable-less seismograph system, which can transmit seismic data in real-time and automatically perform high-precision differential self-positioning. Combining the ZigBee technology with the high-precision differential positioning module, this new seismograph system utilized the wireless personal area network (WPAN) and real-time kinematic (RTK) technologies to improve its on-site performances and to make the field quality control (QC) and self-positioning possible. With the advantages of low-cost, good scalability, and good compatibility, the proposed new cable-less seismograph system can improve the field working efficiency and data processing capability. It has potential applications in noise seismology and mobile seismic monitoring.

## 1. Introduction

Compared with a cabled seismograph, cable-less seismographs have no complicated or bulky cables. As a piece of portable and mobile equipment, a cable-less seismograph is an independent acquisition platform, particularly advantageous when used in difficult terrain, such as mountainous areas or cities, and in areas where cable damage is an issue. Cable-less systems are often promoted as lightweight, logistically simple, and flexible for deployment, and have been widely used in the field of passive source seismic exploration, such as the ambient noise tomography (ANT) [1,2,3], microtremor survey [4,5] and the dense array survey [6,7,8,9]. The main advantages of the cable-less seismograph are casting off the restraint of cables and realizing the array layout in a more flexible way, which can expand the seismic channels infinitely and deploy high-density and wide-azimuth acquisition arrays. A bunch of cables is more susceptible to environmental interference, resulting in a decrease in signal-noise-ratio (SNR) of seismic data [10,11]. In addition, the cost of a cable-less seismograph is low and its miniaturization and portability advantages lie in the fact that it is not necessary to assign a large amount of labor or vehicles to participate in the deployment of the acquisition platform in the fieldwork. Thus, a cable-less seismograph has good mobility and its application can reduce the exploration cost and improve construction efficiency.

**Figure 1:**
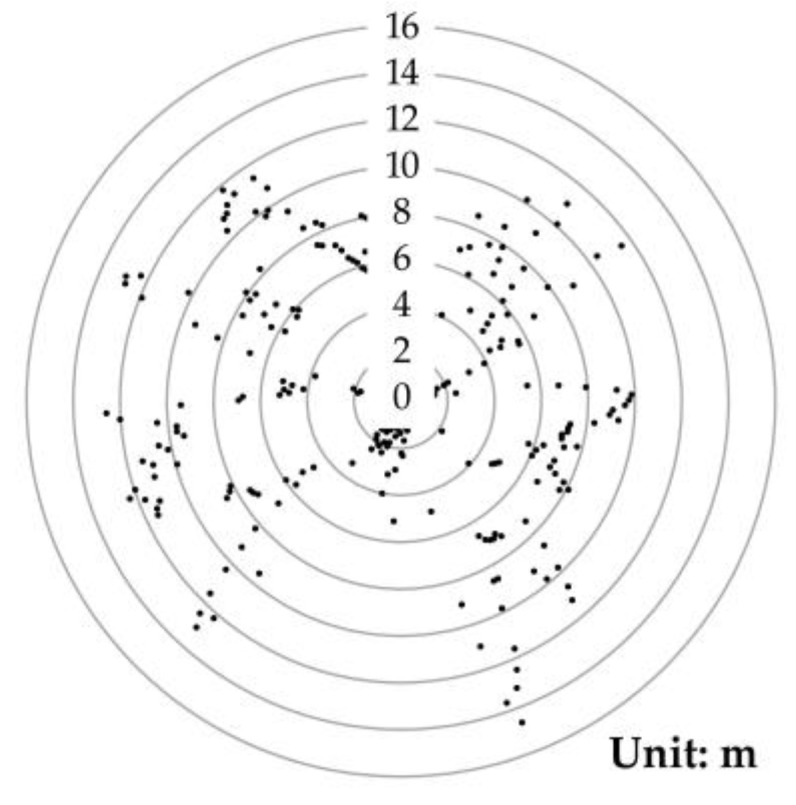
The single point positioning test showed that the accuracy was a 10-m level.

The non-invasive passive-source seismic prospecting technology is insensitive to the distribution of seismic stations and does not need a regular array [12,13,14,15]. In theory, it is possible to arrange seismographs randomly and irregularly in a study area. However, when processing data, the accurate location of each station is essential. Most cable-less seismographs are embedded with the global positioning system (GPS) module so that they can obtain both time service and positioning. As the GPS module uses the single point positioning method, its accuracy might not be high enough to meet the requirements of passive-source seismic prospecting technology. In this study, a location test was carried out to verify the single point positioning accuracy of the existing cable-less seismographs (Figure 1). During the 25 min test, the positioning accuracy was a 10-m level, which was far from meeting the requirements of passive-source seismic prospecting technology.

In order to improve the positioning accuracy, it is necessary to adopt the technology of differential positioning. One way to do this is via the traditional manual single-point-by-point method using expensive special equipment, by which the data processing is not only time-consuming and laborious but also limited by many environmental factors (roads, factories, waters, etc.). Another efficient way is via differential self-positioning, which can reduce the cost of fieldwork and avoid the tedious mapping and positioning work to improve measurement efficiency, which showed great promise in future applications. Liu and Chen [16] investigated the positioning of six seismographs using the network adjustment method and obtained better positioning accuracy through the post-processing of GPS single point location information. However, it was far from the true self-positioning of a seismograph. Zhang [17] used the OEMStar board (produced by NovAtel in Calgary, Alberta, Canada) to perform high-precision and real-time kinematic (RTK) positioning, which can reach a centimeter-level accuracy. However, its positioning method was post-processing and the board was not embedded in the seismograph. Therefore, it is not really self-positioning. Xie and Zhou [18] used the pseudo-range difference (PRD) method to design a differential positioning circuit module with a positioning error smaller than 2 m. The accuracy was not high enough, and the method has not been realized on an integrated instrument. The new seismograph system developed in this study has an embedded high-precision differential positioning module, so that the randomly distributed instruments can achieve a true accurate self-positioning.

At present, many commercial cable-less seismographs are available in the US, European, and Asian countries [19], such as the Unite System (produced by Sercel in Carquefou, France), the Sigma System (produced by iSeis in Ponca City, Oklahoma, USA), the Z-land (produced by Fairfield in Houston, Texas, USA), the GSR/GCX (produced by Geospace in Houston, Texas, USA), the FireFly system (produced by INOVA in Houston, Texas, USA), and several other cable-less seismographs developed by Jilin University, Tongji University, and the Chinese Academy of Sciences in China [20,21]. Most cable-less seismographs record data by self-storage and blind acquisition. In order to ensure the quality of the data collected in the field, it is necessary to evaluate the system consistency among different instruments. However, the consistency test is not a real-time or on-site quality control (QC) procedure. Therefore, even though the “qualified” instrument is selected through the consistency test, there might be drawbacks of bad channels, poor coupling, inaccurate clocks, etc. For traditional blind-recording cable-less seismographs, researchers have studied various wireless technologies to recognize the real-time data transmission, such as the mesh wireless network (MRN) technology of iSeis, cellular network technology of Sercel, and WiFi or 4G technology of Jilin University and the iSeis [22,23,24,25]. The INOVA provides various trace attributes to QC the data, as well as the sensor performance via a very high frequency (VHF) or ultra-high frequency (UHF) radio link. The Sercel uses the 2.4 GHz frequency band, but each unit transmits data individually. Data can either be transmitted in real-time to an antenna or harvested periodically. Because various wireless transmission technologies have their own pros and cons in terms of the cost, stability, and power consumption, the choice of which technology to select is mainly based on the application conditions. Moreover, there are no cable-less seismographs to realize differential positioning.

Considering the cost, stability, power consumption, and other factors, the proposed new cable-less seismograph system in this study was designed using the ZigBee communication technology, which can transmit seismic data stably and rapidly in real-time. The technology makes it convenient for scientific researchers to grasp the working status of each seismic station in real-time. The RTK module embedded in the seismograph used both the GPS and BeiDou Navigation Satellite System (BDS) to achieve an autonomous sub-decimeter level positioning. For the shallow noise exploration (~1 km), the observation radius of the new cable-less seismograph was between tens to hundreds of meters, which met the requirements of the positioning range.

## 2. System Design

### 2.1. Framework

**Figure 2:**
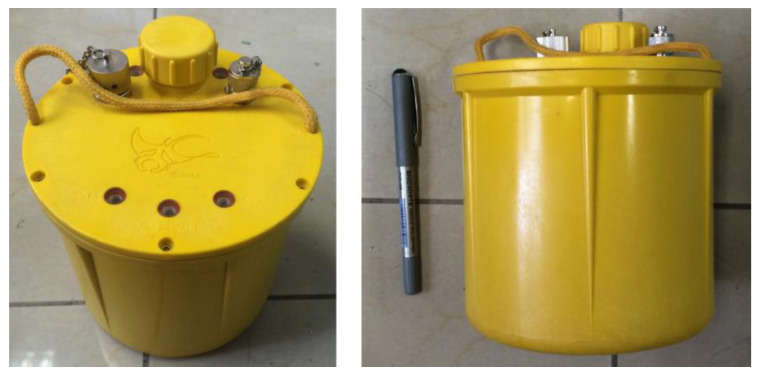
The new cable-less seismograph system is the updated vision of the embedded portable high-precision digital seismograph (EPS), with the functions of real-time data transmitting and differential self-positioning. There is a master node to build a wireless personal area network (WPAN) that enables the EPS to receive differential signals from the global positioning system (GPS) or the BeiDou Navigation Satellite System (BDS) and upload seismic signals.

In this study, the new cable-less seismograph system was created based on the embedded portable high-precision digital seismograph (EPS), manufactured by Chongqing Geological Instrument Factory in Chongqing, China [26,27]. The EPS was a broadband (0.2–200 Hz) micro-power seismograph with a built-in three-component seismic sensor, a BDS and GPS timing and positioning module, an electronic compass, and a rechargeable lithium battery (Figure 2). It was suitable for applications of long-duration unattended monitoring, on-site wide-band seismic observation [28], and the micro-tremor survey [29]. In this study, we used one vertical component geophone for development.

**Figure 3:**
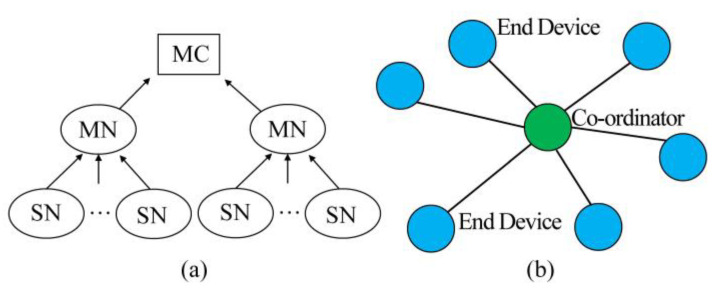
(**a**) The wireless transmission system and (**b**) star topology personal area network. The master node (MN) is the coordinator, connecting with several end devices, which are sensor nodes (SNs).

The ZigBee technology was adopted for the data communication of the new cable-less seismograph system developed in this study. ZigBee has the advantages of low power consumption, low-cost, autonomous networking, and good reliability. For example, when one wireless channel of ZigBee is blocked, other channels can still function to avoid communication interruption. The dynamic routing of ZigBee can automatically scan and find a channel with the shortest distance and smallest interference to maintain reliable data transmission between nodes [30]. The data transmission system (Figure 3) of the new cable-less seismograph system in this study included the master node (MN), sensor node (SN), and master computer (MC). The star network topology was adopted to form personal area networks (PANs). Each PAN had one MN and several SNs, which served as coordinator and end devices, respectively [31].

**Figure 4:**
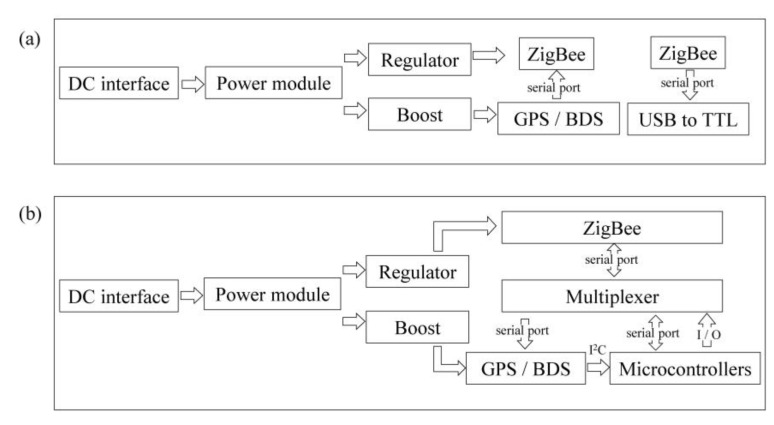
The circuit structures of (**a**) the MN; (**b**) SN. The MN and SN have similar power supply circuit designs, but different ZigBee transmission designs. The MN, which has two ZigBee modules, controls differential data and seismic data communication, respectively, while the SN has one ZigBee module and one multiplexer, which switches the communication with the positioning module and microcontroller unit (MCU).

The MN was designed as a multi-functional electromechanical box, which acted as the manager of the PAN and the base station (BS) of differential positioning, being responsible for the network control, differential message distribution, and seismic data (MiniSeed format) uploading to the MC. The MN had two ZigBee modules (Figure 4a) with different network addresses to identify different PANs. One of them was used to transmit a differential data stream and realize differential positioning; the other was used to transmit MiniSeed blocks to the MC in real-time through a universal serial bus (USB); a transistor–transistor logic (TTL) serial chip. The SN was the EPS with a differential positioning module, a ZigBee transmission module, and a multiplexer (Figure 4b). When the MN was creating a PAN, the SN, as a seismic acquisition station and differential positioning rover station (RS), would find and activate the smoothest channel to access the network, receive differential messages from the MN’s ZigBee for autonomous differential positioning, and transmit SN’s coordinates to the microcontroller unit (MCU) through inter-integrated circuit (I^2^C). After the SN was positioned successfully, the MCU used input/output (IO) interface to control the multiplexer to connect ZigBee, then the seismic data could be transmitted to the MN in real-time.

### 2.2. ZigBee Transmission System

The transmission module of ZigBee used JN5168 wireless microcontrollers (produced by NXP Semiconductor in Eindhoven, Netherlands). The sending, receiving, sleep, and deep sleep current of the JN5168 were 175 mA, 22 mA, 0.7 μA, and 0.1 μA, respectively. The ZigBee module was equipped with a variety of on-chip and off-chip devices, i.e., the analog-to-digital converter (ADC), the I^2^C, a timer, and a serial interface. In order to facilitate the optimization and upgrading of the internal program of the instrument, the ZigBee module used the master input slave output (SPIMISO) for inputting data and adding programs. A reset signal line (RESETN) was used to start the entire reset sequence of the chip. In order to monitor the network working status of the ZigBee module, a red light was added to the general input/output (GPIO0).

### 2.3. Differential Positioning System

The new cable-less seismograph system adopted the RTK differential positioning method, which is a satellite navigation technique used to enhance the precision of position data derived from global navigation satellite systems (GNSS) such as GPS, GLONASS, Galileo, and BeiDou. It uses measurements of the phase of the signal’s carrier wave in addition to the information content of the signal and relies on a base station to provide real-time corrections, providing up to more than centimeter-level accuracy. Therefore, RTK usually needs one BS with accurate known coordinates and several RSs, which are to be positioned. The carrier phase corrections measured by the BS are sent to RSs wirelessly in real-time. The RSs carry out differential processing using their carrier phase values and BS corrections to calculate the precise coordinates of the RSs in real time. RTK has the main advantages of high efficiency, high positioning accuracy, and no error accumulation. Its disadvantage is that the accuracy decreases with the increase of baseline distance. Usually, the baseline length should not exceed 50 km for the cm-level positioning accuracy. The typical field observation distance of a seismograph was less than the kilometer level; therefore, a high precision field self-localization can be realized by the new cable-less seismograph system developed in this study.

For the new cable-less seismograph system developed in this study, the differential positioning module (NEO-M8P) produced by U-BLOX in Thalwil, Switzerland was used. The size of the module was very small (10 mm × 15 mm). The NEO-M8P was composed of an M8P-2 (BS module) and an M8P-0 (RS module). The NEO-M8P module supported both GPS and BDS positioning.

The BS and RS modules of the NEO-M8P were attached to the MN and SN, respectively. The World Geodetic System in 1984 (WGS-84) and the general differential protocol compiled by Radio Technical Commission for Maritime (RTCM) were adopted. When operating, the BS module-generated differential signal was sent to the RS module by the ZigBee system in the form of the RTCM message. The embedded RS module then analyzed the RTCM message and obtained high precision position information.

The voltage of both the BS and RS modules was 5 V. After the BS module on the MN successfully identified or manually defined its location, the RTCM differential data based on the carrier phase observation was distributed to the SN through ZigBee. After receiving the RTCM, the RS module on the SN calculated its location and sent coordinates to an MCU through the I^2^C and to the MN through ZigBee. A pulse per second (PPS) signal from the NEO-M8P was transmitted to the MCU to obtain the current time, which was used on the SN only.

### 2.4. Star Topology PAN

**Figure 5:**
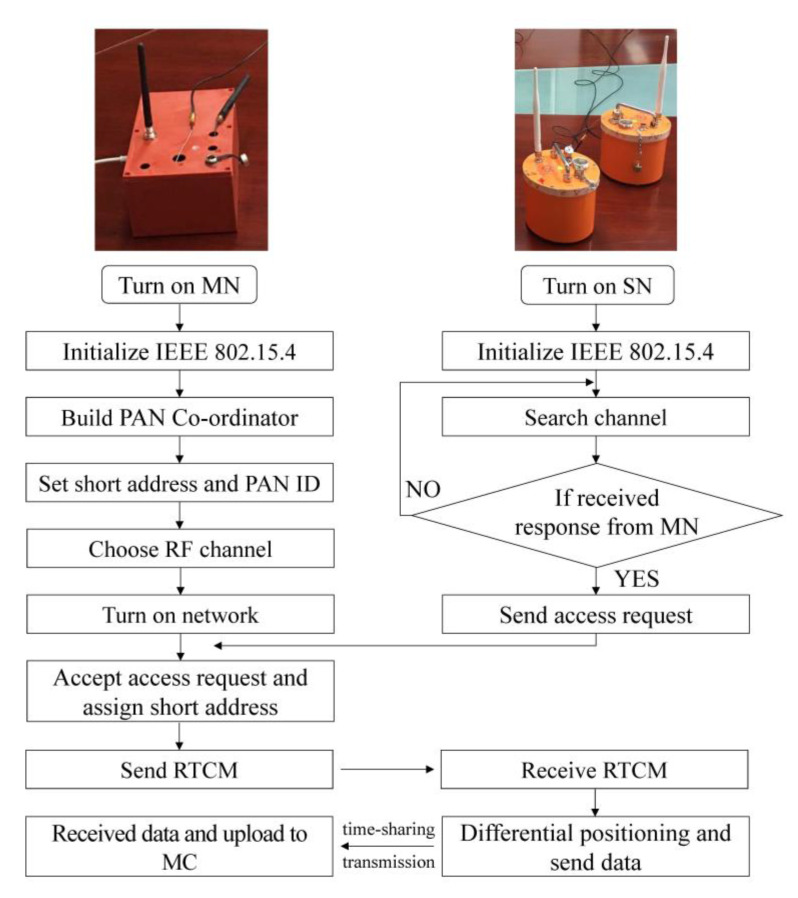
The flow chart of networking for the MN and SN in the frame of the star topology personal area network (PAN).

In this study, the star topology network was used to form PANs. Each PAN had one MN and several SNs (Figure 5). The ZigBee technology used for communications between the MN and SNs was based on the standardized protocol of the Institute of Electrical and Electronics Engineers (IEEE)—IEEE 802.15.4 [32]. The protocol defined the communication standard between adjacent devices by radio frequencies (RFs), which had the characteristics of low power consumption. The physical layer of IEEE 802.15.4 can detect the energy of signal channels and obtain the peak energy of each channel. Therefore, an optimal path can be identified when rebooting. In this study, C language in the Eclipse platform was used to configure the star topology PAN.

The MN is the manager of the PAN controlling the differential message distribution and seismic data transmission. The two ZigBee modules in MN have similar networking processes but different identifications (IDs) to identify different PANs for seismic data transmission and RTCM transmission. The networking process is as follows:

(1) Initialize IEEE 802.15.4 protocol.

(2) Build PAN and coordinator.

(3) Set the short address and distribute the PAN-ID. Using short address to communicate is more convenient and efficient.

(4) Choose RF channel. Energy detection can return the power level for each channel, which can be used for the PAN coordinator to select the radio channel within the specified frequency band.

(5) Turn on the PAN and the coordinator starts to listen for the access request from SNs.

The SNs are responsible for differential positioning and data uploading to the MN, and its networking process is as follows:

(1) Initialize IEEE 802.15.4 protocol.

(2) The SN starts the active channel detection and sends a beacon to find the communication channel with the MN.

(3) After the coordinator agrees to the PAN access request, a unique network short address is assigned to each SN.

## 3. Test of the Developed System

### 3.1. System Operation Test

**Figure 6:**
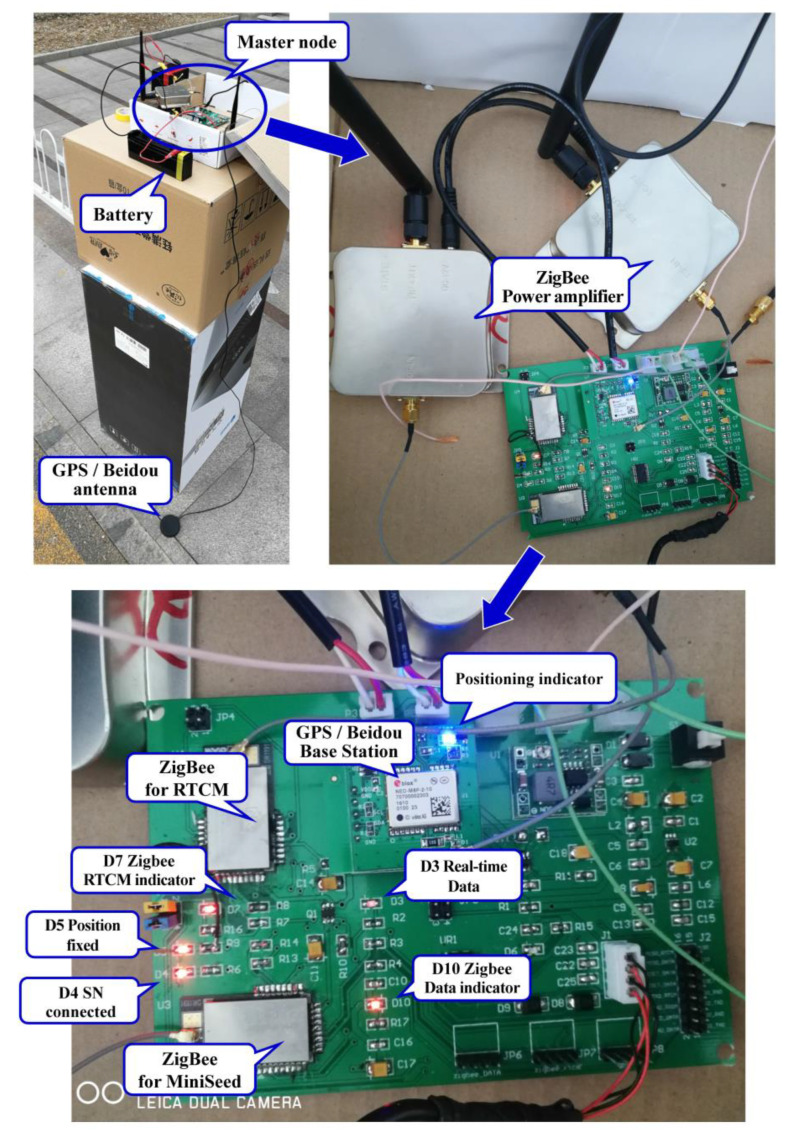
The circuit board and external devices, such as battery, antenna, and power amplifier of the MN.

**Figure 7:**
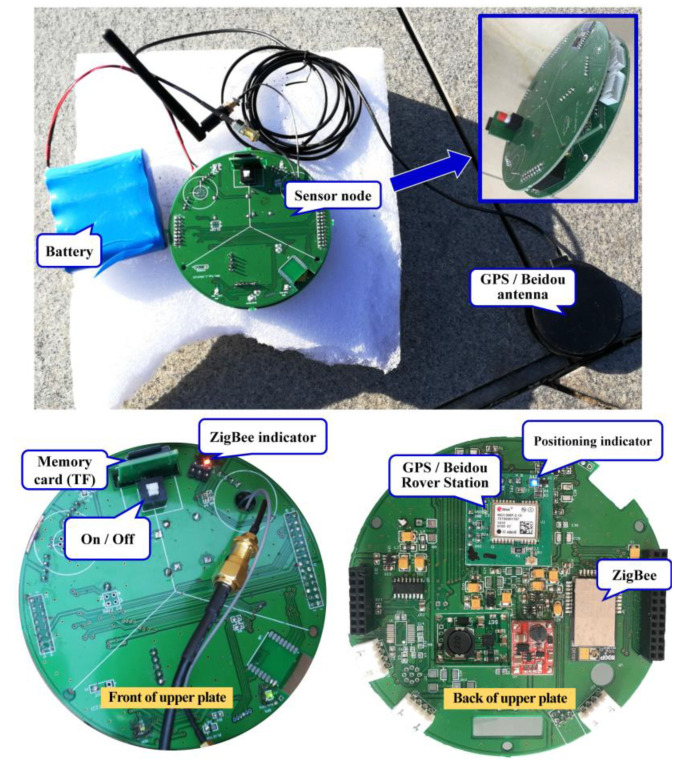
(**a**) The circuit board of the SN has front has two plates; the upper plate for data transmission and the lower plate for data acquisition; (**b**) the front of the upper plate; and (**c**) the back of the upper plate.

While testing the operation of the new cable-less seismograph system, the MN of the system was first turned on and the ZigBee for the RTCM distribution and seismic data transmission were activated. The light of the positioning indicator on the MN turned blue (Figure 6). After the BS module on the MN successfully located the position, RTCM messages were distributed. The SNs of the system were then turned on. When the SN–MN network was successfully communicated, the “ZigBee indicator” light on the SN turned red (Figure 7). The BS module on the MN then started to send the carrier observation and coordinate information to all the SNs through ZigBee, which was responsible for sending the RTCM. After the SNs received the RTCM differential data stream, the RS module on the SNs calculated the precise location information of SNs. The calculated coordinates were then transmitted to the MCU through I^2^C. These coordinates of the SNs were in the National Marine Electronics Association (NMEA) format, which contained the positioning status identification (0-initialization, 1-single point positioning, 2-differential positioning, 4-RTK fixed solution, 5-RTK floating-point solution), longitude, latitude, and elevation (m). When the differential positioning of the SNs was completed, the working status of the ZigBee was switched to seismic data transmission. The geophone on the SNs then recorded seismic data. The recorded seismic data was transmitted to the MCU and stored in a TransFlash (TF) memory card. When the SN was uploading data, another ZigBee module on the MN, which was responsible for MiniSeed data transmission, formed a new PAN after obtaining access permission. In order to ensure stability, the time-sharing data transmission method was used in this study. Finally, the MN successfully transmitted the received data to the MC in real-time through the serial port.

### 3.2. Differential Positioning Performance Test

**Figure 8:**
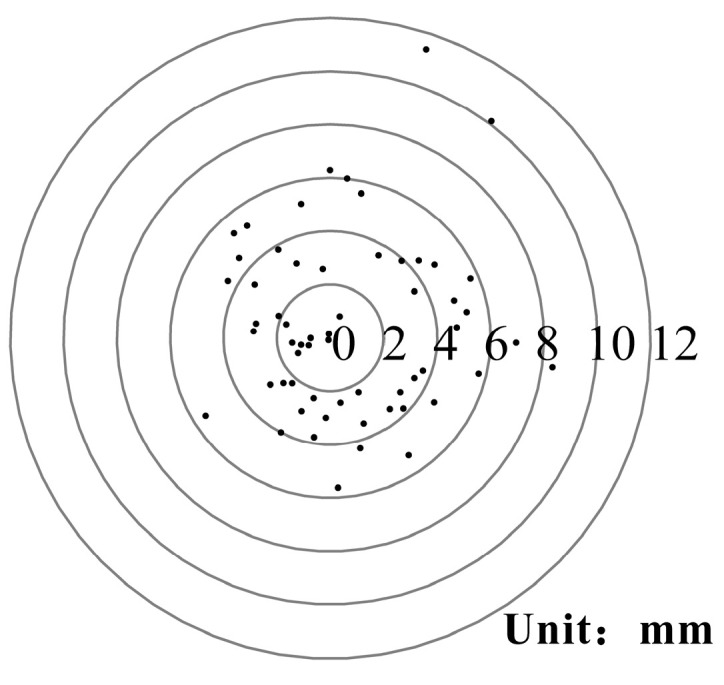
The positioning test of the differential positioning module showed that the accuracy was millimeter-level. The test was carried out in an urban environment, which had a negative impact on the positioning accuracy, resulting in two points at centimeter level.

The performance of the differential positioning was validated by setting a network with a single MN and a single SN, 10 m apart. After the successful positioning of the SN, the latitude and longitude information were recorded 60 times. The arc distance between the SN and the MN was calculated. The distance differences were drafted in a radar format, as shown in Figure 8. It can be seen that the errors of the relative position of the SN and MN were at the mm level. Therefore, high precision self-positioning can be realized using the new cable-less seismograph system.

**Figure 9:**
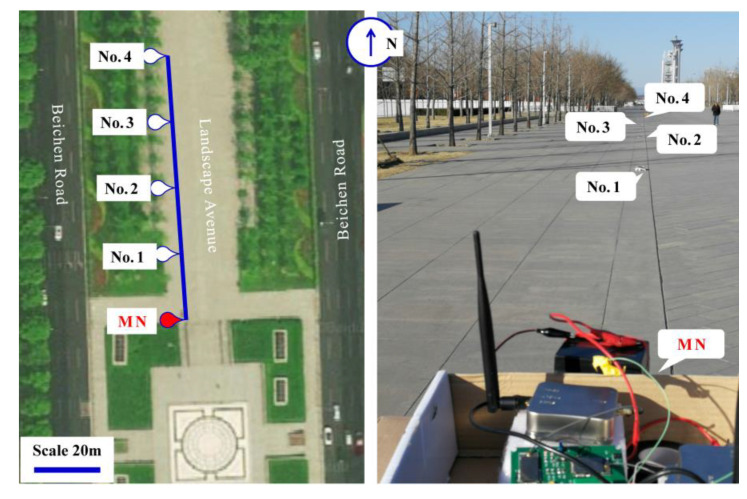
The positioning test site around the urban open land.

In addition to the single MN–SN test, an on-site experiment with a network of a single MN and multiple SNs was conducted on Olympic Landscape Avenue along the axis of Beijing, China (Figure 9). The tested road was approximately 1 km long and 60 m wide. There were no high-rise buildings within a few kilometers on both sides (east and west) of the road. The north of the testing site was an open land area. For this experiment, an 80 m long straight survey line was set with four SNs and one MN at 20 m intervals. The survey line had a 2° angle from the perfect North toward the West.

**Figure 10:**
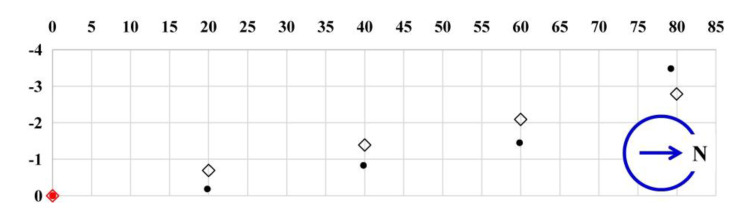
The schematic diagram of the positioning results in the urban open land. The diamonds and dots are the theoretical and measured values, respectively, and the red and black are the MN and SNs, respectively.

During the test, two parameters were used for validating the differential positioning. The first parameter was the errors between the tested coordinates of the SNs and their theoretical values, and the second parameter was the errors of the baseline distances between the SNs (rover station) and the MN (base station). For the first parameter, Table 1 shows the tested latitudes and longitudes of the MN and SNs in the WGS-84 coordinate system. Table 2 shows the horizontal and vertical coordinate differences of the SNs, based on the universal transverse mercator (UTM) projection in meters between theoretical values (*X_mdl_*, *Y_mdl_*) and tested results (*X_UTM_*, *Y_UTM_*). The MN was set as the coordinate origin. Figure 10 is a graphical demonstration of Table 2. For the second parameter, Table 3 shows the actual distances, tested distances, and distance errors between the MN and SNs. The actual distance values in Table 3 were measured by a tape ruler. The tested distances in the table were the calculated arc lengths based on the WGS-84 coordinate system.

It can be seen that the errors between the measured positions of SNs and their actual positions were positively correlated with the baseline lengths. The errors were approximately 0.53 m for a 20 m distance, 0.58 m for a 40 m distance, 0.66 m for a 60 m distance, and 1.02 m for an 80 m distance. The error rates were in a range of 1%–2.5%. This positioning accuracy was within a decimeter level, as required for an urban and open land area.

### 3.3. Data Transmission Performance Test

There are two communication protocols in the real-time data transmission system: data protocol and time stamp protocol. The data protocol format is as follows:

“3A”, “11”, “54”, “data body”, “frame”, “Sn’s ID”, “3B”

The first three bytes are the start character, length identifier, and data type character, followed by the data body and the frame, a count variable. The last is the termination character with Sn’s ID before it.

The format of time protocol is as follows:

“3A”, “OC”, “99”, “s, min, h, day, month, year, ms”, “Sn’s ID”, “3B”

Similar to the data protocol, each time stamp has the first three bytes of the start character, length identifier, and time type character, followed by the second, minute, hour, day, month, year, and millisecond. The last two are the Sn’s ID and the termination character.

In order to control the SNs by MC, four command protocols are defined, as follows:

To open data acquisition (AD): “3A”, “03”, “52”, “Sn’s ID”.

To close AD: “3A”, “03”, “53”, “Sn’s ID”.

To open data transmission: “3A”, “03”, “54”, “Sn’s ID”.

To close data transmission: “3A”, “03”, “55”, “Sn’s ID”.

**Figure 11:**
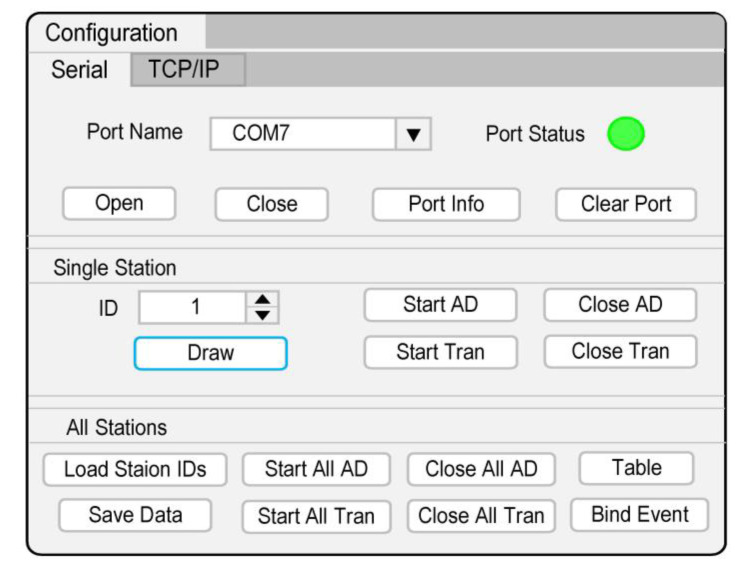
The MC console.

The MC console has two preset modes of serial port and network port communication. The network port uses the transmission control protocol/internet protocol (TCP/IP). Taking the actual serial port used in this study as an example, it can switch serial port and display port information. The control functions for a single SN include selecting Sn’s ID, turning on/off AD, turning on/off data transmission (Tran), and displaying real-time waveform (Draw). The control functions for multiple SNs include importing Sns’ ID list, turning on/off AD, turning on/off Tran for all of SNs, and saving data (Figure 11).

For the data transmission performance test in this study, the seismic data acquisition and transmission started after all single SNs were self-positioned. The data transmission rate was about 2 KB/s and the sampling rate was 100 Hz. The SNs designed in this study adopted the time-sharing method to transmit real-time seismic data, which avoided the defect, which is that the ZigBee module cannot send and receive data at the same time. This way, the MC would not stop working because of any conflict between the upstream data channel and the downstream command channel.

**Figure 12:**
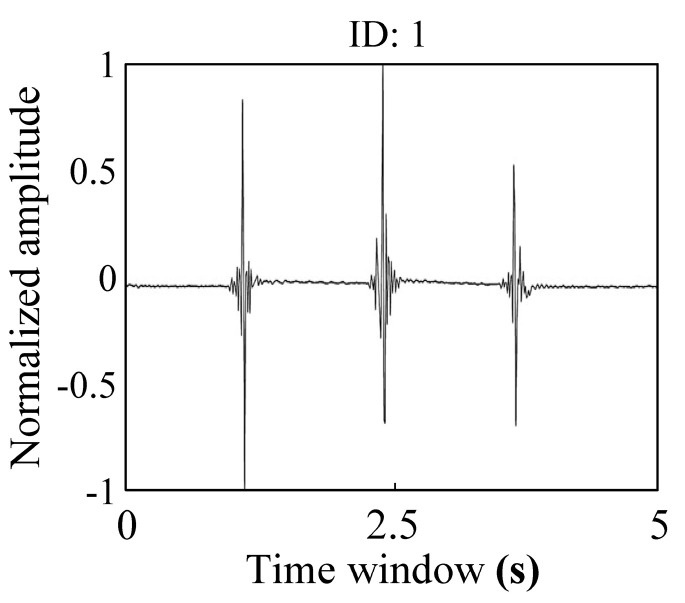
The real-time seismic waveform recorded by a single sensor node.

Figure 12 shows the real-time seismic data example recorded by a single SN. Based on 30 min of continuous measurement, the stability of the ZigBee wireless transmission of the new cable-less seismograph system in this study was validated.

**Figure 13:**
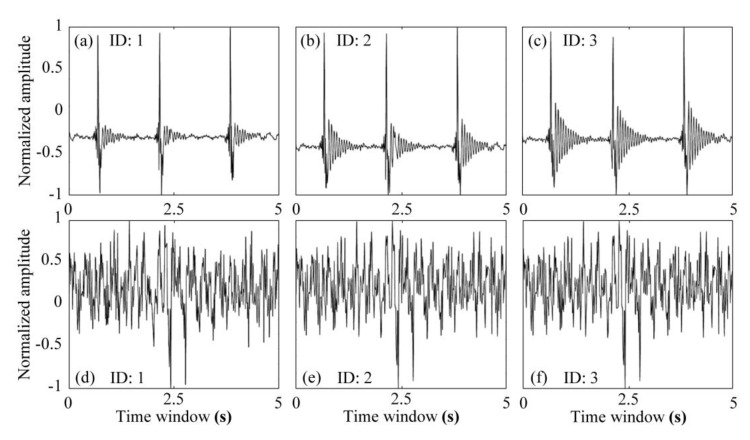
The real-time seismic waveform recorded by three sensor nodes. (**a**–**c**) vibration induced by knocking and (**d**–**f**) ambient noise.

For the multi-SNs test of this study, five SNs were connected to an MN at the same time, which formed a star topology PAN. Figure 13 shows three recorded real-time seismic waveforms, which were consistent.

### 3.4. Configuration and Parameter

The new cable-less seismograph system developed in this study included the main node (MN) and sensor nodes (SNs). Based on the system testing discussed in previous sections, their parameters and cost were summarized in Table 4.

## 4. Discussion

The new cable-less seismograph system is equipped with a 24-bit ADC and a single vertical geophone. According to the 2 KB/s transmission rate of ZigBee and the 100 Hz sampling rate, it was estimated that six SNs in maximum could be supported by the system. More SNs may cause data redundancy or loss. However, the number of SNs can be expanded by reducing the sampling rate or increasing the number of MNs. The transmission distance of the system could be improved as well. The transmission distance of the antenna, shown in Figure 5, was about 300 m, which met the requirement of the shallow seismic exploration with passive source. For deep exploration, a 2.4 G directional high-gain planar antenna can be used to significantly improve the transmission distance to about 1 km. In order to ensure that the MN’s wireless signals (RTCM message distribution and seismic signal reception) cover all the SNs and that the PAN could provide stable networking and communication, the MN could adopt an omnidirectional antenna and a power amplifier with the highest setting to enhance the “one-to-many” transmission capacity and the SNs could adopt the 2.4 G directional high-gain planar antenna to enhance the “one-to-one” communication capability. In addition, a MC software with functions of QC based on SNR and map display through an application programming interface (API) is being developed. In order to meet the needs of different users and application scenarios, other technological methods for data transmission, such as Wi-Fi and 4G, are being conducted.

## 5. Conclusions

For the passive source seismic exploration, a new cable-less seismic acquisition system was developed in this study. Based on the ZigBee wireless transmission technology with the framework of star topology wireless PAN, the new system can transmit seismic data stably. The working status of the system can be monitored in real-time and the high-precision self-positioning can be realized by the system using the carrier phase differential positioning.

The MN of the system was designed as a multi-functional electromechanical box, which acted as a manager and base station. It was responsible for the PAN control, RTCM distribution, and seismic data uploading to the master computer. The SN of the system, namely cable-less seismograph, which acted as an acquisition and rover station, was responsible for the differential positioning and seismic data transmission to the MN.

The results of the positioning test in this study showed that the differential self-positioning module can achieve a millimeter level accuracy, which was much better than the required decimeter level accuracy for urban and open land areas. The data transmission validation test results in this study showed that the wireless system based on ZigBee technology can realize the real-time data transmission stably and reliably.

## Figures and Tables

**Table 1:** The tested results around the urban open land.

Station	Latitude	Longitude
**Base**	39°58′37.2199″	116°23′16.1974″
**No. 1**	39°58′39.7879″	116°23′16.0277″
**No. 2**	39°58′37.8631″	116°23′16.1838″
**No. 3**	39°58′38.5111″	116°23′16.1508″
**No. 4**	39° 58′39.1602″	116° 23′16.1190″

**Table 2:** The positioning accuracy around the urban open land: coordinate.

Station	*X_UTM_*	*Y_UTM_*	*X_mdl_*	*Y_mdl_*	*△X*	*△Y*	Error ^1^
**Base**	0	0	0	0	0	0	0
**No. 1**	−0.189	19.832	−0.698	19.988	0.156	0.509	0.532
**No. 2**	−0.833	39.817	−1.396	39.976	0.159	0.563	0.585
**No. 3**	−1.451	59.833	−2.094	59.963	0.130	0.643	0.656
**No. 4**	−3.482	79.203	−2.792	79.951	0.748	0.690	1.018

^1^ Error = sqrt (*△X*^2^ + *△Y*^2^).

**Table 3:** The positioning accuracy around the urban open land: distance.

Station	Base	No. 1	No. 2	No. 3	No. 4
**Base**	0 (0) 0	20.000 (19.841) 0.159	40.000 (39.841) 0.159	60.000 (59.873) 0.127	80.000 (79.308) 0.692
**No. 1**	20.000 (19.841) 0.159	0 (0) 0	20.000 (20.003) 0.003	40.000 (40.035) 0.035	60.000 (59.483) 0.517
**No. 2**	40.000 (39.841) 0.159	20.000 (20.003) 0.003	0 (0) 0	20.000 (20.033) 0.033	40.000 (39.489) 0.511
**No. 3**	60.000 (59.873) 0.127	40.000 (40.035) 0.035	20.000 (20.033) 0.033	0 (0) 0	20.000 (19.483) 0.517
**No. 4**	80.000 (79.308) 0.692	60.000 (59.483) 0.517	40.000 (39.489) 0.511	20.000 (19.483) 0.517	0 (0) 0

**Table 4:** Configuration, parameter, and cost.

Index	Master Node	Sensor Node
Size	250 mm × 330 mm × 110 mm	Φ 140 × 170 mm
Weight	6.2 kg (including battery)	2.3 kg (including battery)
Power	5 V DC	5-12 V DC
ADC	–	24-bit
Wireless method	ZigBee	ZigBee
Geophone	–	Vertical moving-coil velocity
Frequency band	–	5 s–200 Hz
Sampling rate	–	50–1000 Hz
Power consumption	Positioning: 0.38 w Transmitting: 0.64 w	Positioning: 0.38 w Transmitting: 1.00 w
Positioning accuracy	–	<2% of baseline length
Transmission speed	2 KB/s	2 KB/s
PAN capacity	1 MN and 6 SNs with 100 sps	
ZigBee price	$32.0	$16.0
M8P price	$199.0	$199.0
